# Novel Copper Complexes as Visible Light Photoinitiators for the Synthesis of Interpenetrating Polymer Networks (IPNs)

**DOI:** 10.3390/polym14101998

**Published:** 2022-05-13

**Authors:** Mahmoud Rahal, Guillaume Noirbent, Bernadette Graff, Joumana Toufaily, Tayssir Hamieh, Didier Gigmes, Frédéric Dumur, Jacques Lalevée

**Affiliations:** 1 Université de Haute-Alsace, CNRS, IS2M UMR 7361, F-68100 Mulhouse, France; mahmoud-rahal@outlook.com (M.R.); bernadette.graff@uha.fr (B.G.); 2 Université de Strasbourg, F-67087 Strasbourg, France; 3 Laboratory of Materials, Catalysis, Environment and Analytical Methods (MCEMA) and LEADDER Laboratory, Faculty of Sciences, Doctoral School of Sciences and Technology (EDST), Lebanese University, Beirut 6573-14, Lebanon; joumana.toufaily@ul.edu.lb (J.T.); tayssir.hamieh@ul.edu.lb (T.H.); 4 Aix Marseille Univ, CNRS, ICR UMR 7273, F-13397 Marseille, France; guillaume.noirbent@outlook.fr (G.N.); didier.gigmes@univ-amu.fr (D.G.)

**Keywords:** copper complex, photocomposite, LED, laser write, free radical photopolymerization

## Abstract

This work is devoted to the study of two copper complexes (Cu) bearing pyridine ligands, which were synthesized, evaluated and tested as new visible light photoinitiators for the free radical photopolymerization (FRP) of acrylates functional groups in thick and thin samples upon light-emitting diodes (LED) at 405 and 455 nm irradiation. These latter wavelengths are considered to be safe to produce polymer materials. The photoinitiation abilities of these organometallic compounds were evaluated in combination with an iodonium (Iod) salt and/or amine (e.g., *N*-phenylglycine—NPG). Interestingly, high final conversions and high polymerization rates were obtained for both compounds using two and three-component photoinitiating systems (Cu1 (or Cu2)/Iodonium salt (Iod) (0.1%/1% *w*/*w*) and Cu1 (or Cu2)/Iod/amine (0.1%/1%/1% *w*/*w*/*w*)). The new proposed copper complexes were also used for direct laser write experiments involving a laser diode at 405 nm, and for the photocomposite synthesis with glass fibers using a UV-conveyor at 395 nm. To explain the obtained polymerization results, different methods and characterization techniques were used: steady-state photolysis, real-time Fourier transform infrared spectroscopy (RT-FTIR), emission spectroscopy and cyclic voltammetry.

## 1. Introduction

The elaboration of polymers by photochemical means, such as free radical photopolymerization (FRP) and cationic photopolymerization (CP), have been mainly based on the use of metal-free organic dyes and photoinitiators at the industrial and academic levels [1,2,3,4,5,6,7,8,9,10,11,12], and these synthetic processes (FRP and CP) are widely used in different fields, e.g., dentistry [13,14,15,16,17,18,19,20,21,22,23], adhesives [24,25,26,27,28], coatings [29,30,31,32,33], composites [34], medicine [35,36,37,38,39,40], direct laser write, 3D and 4D printing [41,42,43,44,45,46,47,48,49,50], etc. On the other hand, organometallic compounds are not really used in industry; in other words, manufacturers avoid incorporating metallic compounds in their synthetic formulations due to their potential toxicity and price [51,52,53,54,55,56,57,58,59,60,61,62,63,64]. With their photochemical properties, such as high-absorption properties in the near-UV and visible range [65,66,67,68], long-lived excited states [69,70,71,72,73,74], suitable redox potentials [75,76,77,78,79,80,81,82,83,84,85,86,87,88,89], copper complexes can be used as photoinitiators (PIs)/photoredox catalysts able to produce active species, according to a catalytic cycle [90,91]. Therefore, it is very important to develop new metal-free photoinitiators or low-cost organometallic-based complexes [92,93,94,95].

In fact, copper complexes have attracted much attention and intense efforts have been devoted in recent years to the development of new copper complexes of improved photosensitivity, due to their competitive costs compared to other metal complexes. Copper complexes bearing a pyridine-based chelate ligand showed excellent photochemical properties for photocatalysis process, such as high-oxidation potential in the excited state [96,97,98,99], long-excited-state lifetime, high-emission quantum yields and high-absorption properties in the UV-visible region. Furthermore, copper complex derivatives have already been tested as PIs for FRP, CP, as well as IPN synthesis [100,101,102,103,104].

**Figure 1:**
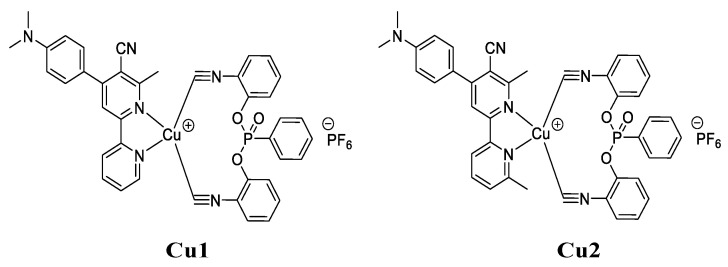
Copper complexes used in this work as PIs.

In this paper, two new copper complexes (Cu1–Cu2) (Figure 1) were synthesized and investigated as visible light photoinitiators upon exposure to LEDs at 405 and 455 nm for FRP, CP and the synthesis of interpenetrating polymer networks (IPNs) of acrylate/epoxy monomer blends. These compounds will be incorporated in two (Cu1 (or Cu2)/Iod (0.1%/1% *w*/*w*)) and three-component (Cu1 (or Cu2)/Iod/NPG (0.1%/1%/1% *w*/*w*/*w*)) photoinitiating systems (PISs) to produce polymer materials by free radical photopolymerization and the polymerization of acrylate/epoxy blend (IPNs). The photoinitiating ability of copper complexes will also be explained based on the interaction of Cu1 (or Cu2)/Iod and Cu1 (or Cu2)/Iod/NPG, which can be studied using different techniques and characterization processes, e.g., steady-state photolysis, cyclic voltammetry, fluorescence quenching and electron spin resonance spin trapping. Finally, to demonstrate the effectiveness of these new copper complex-based photoinitiators, experiments using direct laser writing (DLW), 3D printing and photocomposites synthesis were carried out in this work using different irradiation sources.

## 2. Materials and Methods

### 2.1. Synthesis of Chalcones, Ligands and Copper Complexes

Experimental conditions and acquisition conditions have been detailed elsewhere [13,92,100]. The two chalcones used for the design of ligands L1 and L2 were then engaged in a cyclization reaction with β-aminocrotonitrile according to a reaction reported in 1992 by Masaki Matsui [105,106]. *Bis*(2-isocyanophenyl) phenylphosphonate (binc) was synthesized by adapting a literature procedure [107,108].

*Synthesis of bis(2-isocyanophenyl) phenylphosphonate (binc)*


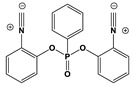


Benzoxazole (9.1 g, 76.3 mmol, 1.0 equiv.) was dissolved in dry THF (200 mL). The solution was cooled at −78 °C and *n*-BuLi (2.5 M in hexane, 32.0 mL, 80.0 mmol, 1.05 equiv.) was added. Stirring was maintained for 1.5 h at this temperature. Phenyl phosphonic dichloride (5.7 mL, 4.04 mmol, 0.53 equiv.) was added and the solution could warm to room temperature. The solution was poured in Et_2_O:NaHCO_3_ (2:1, 150 mL). The organic phase was washed with water several times, dried over magnesium sulfate and the solvent removed under reduced pressure. The residue was crystallized in pentane/ethyl acetate (4/1) to provide the ligand (55% yield) as a light brown solid. ^1^H NMR (400 MHz, CDCl_3_) δ(ppm): 8.23–8.11 (m, 2H), 7.69 (td, *J* = 7.4, 1.3 Hz, 1H), 7.58 (dt, *J* = 12.5, 6.3 Hz, 2H), 7.48 (d, *J* = 8.4 Hz, 2H), 7.39 (d, *J* = 7.9 Hz, 2H), 7.34 (td, *J* = 8.1, 1.6 Hz, 2H), 7.18 (t, *J* = 7.7 Hz, 2H); ^13^C NMR (101 MHz, CDCl_3_) δ(ppm): 169.7, 145.7, 145.7, 134.5, 132.9, 132.8, 130.7, 130.7, 129.3, 129.1, 128.3, 125.9, 124.1, 124.1, 121.7, 121.7. (isonitrile carbons not detected); HRMS (ESI MS) *m*/*z*: theor: 360.0664 found: 360.0666 (M^+^. detected).

*Synthesis of (E)-3-(4-(dimethylamino)phenyl)-1-(pyridin-2-yl)prop-2-en-1-one*


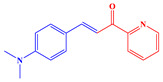


Next, 4-(Dimethylamino)benzaldehyde (1.49 g, 10.0 mmol, M = 149.19 g/mol) and 1-(pyridin-2-yl)ethan-1-one (1.21 g, 10.0 mmol, M = 121.14 g/mol) were suspended in ethanol (50 mL) and aq. KOH (40%) (10 mL) was added. After stirring overnight, the solid was filtered off, washed with ethanol and water, and dried under vacuum. The product was purified by filtration on a plug of SiO_2_ using dichloromethane (DCM) as the eluent (2.32 g, 92% yield). ^1^H NMR (400 MHz, CDCl_3_) δ(ppm): 8.61–8.49 (m, 1H), 8.01 (d, *J* = 7.4 Hz, 1H), 7.92 (d, *J* = 15.8 Hz, 1H), 7.77 (d, *J* = 15.8 Hz, 1H), 7.66 (t, *J* = 7.0 Hz, 1H), 7.45 (d, *J* = 7.7 Hz, 2H), 7.31–7.22 (m, 1H), 6.49 (d, *J* = 7.8 Hz, 2H), 2.83 (s, 6H); ^13^C NMR (101 MHz, CDCl_3_) δ(ppm): 189.16, 155.00, 152.11, 148.69, 145.94, 136.89, 130.90, 126.40, 122.99, 122.72, 115.47, 111.74, 40.06; HRMS (ESI MS) *m*/*z*: theor: 253.1296 found: 253.1299 ([M + H]^+^ detected).

*Synthesis of (E)-3-(4-(dimethylamino)phenyl)-1-(6-methylpyridin-2-yl)prop-2-en-1-one*


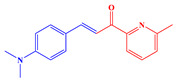


Following this, 4-(Dimethylamino)benzaldehyde (2.21 g, 14.8 mmol, M = 149.19 g/mol) and 1-(6-methylpyridin-2-yl)ethan-1-one (2.00 g, 14.8 mmol, M = 135.17 g/mol) were dissolved in ethanol (50 mL) and aq. KOH (40%) (15 mL) was added. After stirring the solution overnight, the resulting solid was filtered off. It was purified by filtration on a plug of SiO_2_ using DCM as the eluent. For a higher purity, the solid was first dissolved in DCM and precipitated by addition of pentane (3.51 g, 89% yield). ^1^H NMR (400 MHz, CDCl_3_) δ(ppm): 8.02 (d, *J* = 15.8 Hz, 1H), 7.90 (d, *J* = 7.5 Hz, 1H), 7.84 (d, *J* = 15.9 Hz, 1H), 7.65 (t, *J* = 7.7 Hz, 1H), 7.58–7.53 (m, 2H), 7.23 (d, *J* = 7.4 Hz, 1H), 6.66–6.60 (m, 2H), 2.97 (s, 6H), 2.60 (s, 3H); ^13^C NMR (101 MHz, CDCl_3_) δ(ppm): 189.62, 157.67, 154.57, 152.04, 145.63, 136.95, 130.84, 126.02, 123.26, 119.84, 115.94, 111.77, 40.13, 24.54; HRMS (ESI MS) *m*/*z*: theor: 267.1453 found: 267.1451 ([M + H]^+^ detected).

*Synthesis of 4-(4-(dimethylamino)phenyl)-6-methyl-[2,2′-bipyridine]-5-carbonitrile*


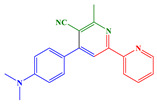


Potassium *tert*-butoxide (1.2 g) and β-aminocrotonitrile (4.92 g, 60 mmol, M = 82.10 g/mol) were dissolved in acetonitrile (300 mL) and the solution was heated at 35 °C for 15 min. Chalcone (*E*)-3-(4-(dimethylamino)phenyl)-1-(pyridin-2-yl)prop-2-en-1-one (2.52 g, 10.0 mmol, M = 252.32 g/mol) was added and stirring was maintained for three days. The solid was filtered off and washed with ethanol and water. It was purified by filtration on a plug of SiO_2_ using DCM as the eluent (2.83 g, 90% yield). ^1^H NMR (400 MHz, CDCl_3_) δ(ppm): 8.70 (ddd, *J* = 4.8, 1.7, 0.9 Hz, 1H), 8.50 (dt, *J* = 8.0, 1.0 Hz, 1H), 8.40 (s, 1H), 7.85 (td, *J* = 7.8, 1.8 Hz, 1H), 7.72–7.64 (m, 2H), 7.36 (ddd, *J* = 7.5, 4.8, 1.2 Hz, 1H), 6.85–6.77 (m, 2H), 3.05 (s, 6H), 2.89 (s, 3H); ^13^C NMR (101 MHz, CDCl_3_) δ(ppm): 162.47, 157.15, 155.03, 154.08, 151.47, 149.38, 137.02, 129.74, 129.74, 124.47, 123.44, 122.10, 118.22, 117.68, 111.97, 111.97, 106.10, 40.19, 40.19, 24.38; HRMS (ESI MS) *m*/*z*: theor: 315.1565 found: 315.1564 ([M + H]^+^ detected).

*Synthesis of 4-(4-(dimethylamino)phenyl)-6,6′-methyl-[2,2* *′-bipyridine]-5-carbonitrile*


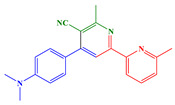


Potassium *tert*-butoxide (1.2 g) and β-aminocrotonitrile (4.92 g, 60 mmol, M = 82.10 g/mol) were dissolved in acetonitrile (300 mL) and the solution was heated at 35 °C for 15 min. Chalcone (*E*)-3-(4-(dimethylamino)phenyl)-1-(6-methylpyridin-2-yl)prop-2-en-1-one (L3) (2.66 g, 10.0 mmol, M = 266.34 g/mol) was added and stirring was maintained for three days. The solid was filtered off and washed with ethanol and water. It was purified by filtration on a plug of SiO_2_ using DCM as the eluent (2.76 g, 84% yield). ^1^H NMR (400 MHz, CDCl_3_) δ(ppm): 8.40 (s, 1H), 8.27 (d, *J* = 7.8 Hz, 1H), 7.72 (t, *J* = 7.8 Hz, 1H), 7.69–7.63 (m, 2H), 7.21 (d, *J* = 7.6 Hz, 1H), 6.85–6.79 (m, 2H), 3.05 (s, 6H), 2.88 (s, 3H), 2.63 (s, 3H); ^13^C NMR (101 MHz, CDCl_3_) δ(ppm): 162.36, 158.24, 157.56, 154.37, 154.00, 151.43, 137.13, 129.70, 129.70, 124.09, 123.68, 119.11, 118.27, 117.78, 112.01, 112.01, 105.95, 40.21, 40.21, 24.61, 24.38; HRMS (ESI MS) *m*/*z*: theor: 329.1722 found: 329.1719 ([M + H]+ detected).

*Synthesis of Cu1*


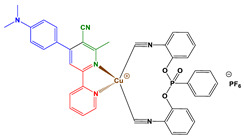


Tetrakis(acetonitrile)copper(I) hexafluorophosphate (372 mg, 1.0 mmol, M = 372.72 g/mol), *bis*(2-isocyanophenyl)phenylphosphonate (binc) (360 mg, 1.0 mmol, M = 360.31 g/mol) and 4-(4-(dimethylamino)phenyl)-6-methyl-[2,2′-bipyridine]-5-carbonitrile (314 mg, 1.0 mmol, M = 314.39 g/mol) were dissolved in DCM (100 mL) and the solution was stirred at 25 °C for 2 h. The solution was concentrated to ca. 5 mL. Diethyl ether was added, as the product was a solid (866 mg, 98% yield). ^1^H NMR (400 MHz, CDCl_3_) δ(ppm): 8.96 (s, 1H), 8.51 (s, 1H), 8.26 (s, 1H), 8.14 (dd, *J* = 14.2, 7.6 Hz, 2H), 7.85–7.70 (m, 4H), 7.70–7.59 (m, 4H), 7.52–7.35 (m, 5H), 7.24 (t, *J* = 8.1 Hz, 2H), 6.94 (s (br), 2H), 3.21 (s, 3H), 3.11 (s, 6H); HRMS (ESI MS) *m*/*z*: theor: 737.1486 found: 737.1481 (M^+^. detected); Anal. Calc. for C_40_H_31_CuF_6_N_6_O_3_P_2_: C, 54.4; H, 3.5; O, 5.4; Found: C, 54.6; H, 3.4; O, 5.5%.

*Synthesis of Cu2*


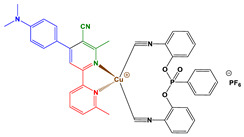


Tetrakis(acetonitrile)copper(I) hexafluorophosphate (372 mg, 1.0 mmol, M = 372.72 g/mol), *bis*(2-isocyanophenyl)phenyl phosphonate (binc) (360 mg, 1.0 mmol, M = 360.31 g/mol) and 4-(4-(dimethylamino)phenyl)-6,6′-methyl-[2,2′-bipyridine]-5-carbonitrile (328 mg, 1.0 mmol, M = 328.42 g/mol) were dissolved in DCM (100 mL) and the solution was stirred at 25 °C for 2 h. The solution was concentrated to ca. 5 mL. Addition of diethyl ether precipitated the product as a yellow solid (883 mg, 98% yield). ^1^H NMR (400 MHz, DMSO) δ(ppm): 8.76 (s, 1H), 8.62 (s, 1H), 8.21 (s, 1H), 8.02 (dd, *J* = 13.2, 7.2 Hz, 2H), 7.79 (s, 6H), 7.67 (s, 2H), 7.56 (t, *J* = 7.9 Hz, 2H), 7.35 (dd, *J* = 21.3, 8.0 Hz, 5H), 6.92 (d, *J* = 8.8 Hz, 2H), 3.14 (s, 3H), 3.06 (s, 6H), 2.93 (s, 3H); HRMS (ESI MS) *m*/*z*: theor: 751.1642 found: 751.1639 (M^+^. detected); Anal. Calc. for C_41_H_33_CuF_6_N_6_O_3_P_2_: C, 54.9; H, 3.7; O, 5.3; Found: C, 55.0; H, 3.4; O, 5.5%.

### 2.2. Other Chemicals

**Figure 2:**
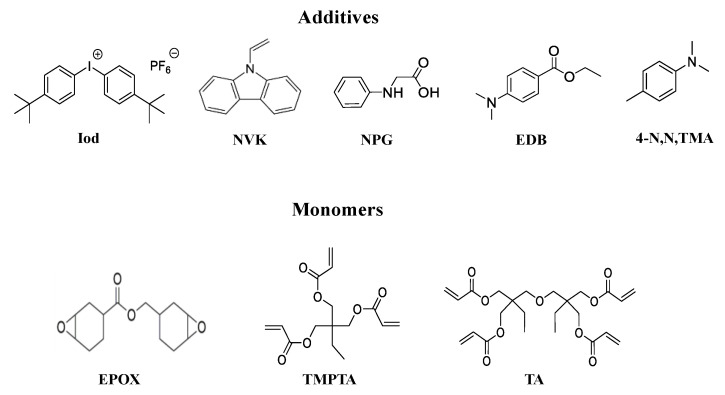
Other organic compounds used.

Chemical structure of the different monomers and additives are presented in Figure 2. Di-*tert*-butyl-diphenyl iodonium hexafluorophosphate (Iod) and ethyl 4-(dimethylamino)benzoate (EDB) were obtained from Lambson Ltd. (UK). Di(trimethylolpropane) tetraacrylate (TA), trimethylolpropane triacrylate (TMPTA), (3,4-epoxycyclohexane)methyl 3,4-epoxycyclohexylcarboxylate (EPOX; Uvacure 1500), *N*-phenylglycine (NPG), *N*-vinylcarbazole (NVK) and *N,N*-dimethyl-*p*-toluidine (TMA) were obtained from Allnex or Sigma Aldrich. TA, TMPTA and EPOX were chosen as acrylic and cationic monomers for the radical and cationic polymerizations.

### 2.3. Irradiation Sources: Light-Emitting Diodes

All the irradiation sources used during these experiments are based on light-emitting diodes (LEDs) and used as safe sources: (1) LED at 375 nm (I_0_ = 75 mW·cm^−2^) for the photolysis experiments, (2) LED at 405 nm (I_0_ = 110 mW·cm^−2^) and 455 nm (I_0_ = 75 mW·cm^−2^) for the photopolymerization experiments, (3) LED conveyor at 395 nm (I_0_ = 4 W·cm^−2^) for the photocomposite synthesis.

### 2.4. Photopolymerization Kinetics Determination by Real-Time Fourier Transform Infrared Spectroscopy (RT-FTIR)

In the present work, copper derivatives were used in two and three-component PISs for both FRP and CP under irradiation with LEDs at 405 and 455 nm. PISs were mainly based on two-component Cu1 (or Cu2)/Iod (0.1%-0.2%-0.5%/1% *w*/*w*) and three-component Cu1 (or Cu2)/Iod/amine (NPG, NVK) (0.1%/1%/1% *w*/*w*/*w*) systems. The weight percOKent of the photoinitiating (PI, co-initiator and amine) system was calculated from the global monomer content. Firstly, two different samples were studied for each photosensitive formulation in (i) thick (thickness = 1.4 mm) and (ii) thin samples (thickness = 25 µm). The epoxy and acrylate conversions were continuously followed by RT-FTIR using a JASCO FTIR 6600 (JASCO France, Lisses, France), so it was possible to determine the final conversion of reactive functions and to calculate the polymerization rate of each kinetic. Acrylate functions in thick and thin samples show peaks towards 6160 cm^−1^ and 1630 cm^−1^, respectively, and the epoxide functions show peaks around 3600 cm^−1^ and 790 cm^−1^ for the thick and thin samples, respectively.

### 2.5. Redox Potentials: Electrochemical Properties

Redox potentials of copper derivatives were determined in DCM by cyclic voltammetry using tetrabutylammonium hexafluorophosphate as the supporting electrolyte (potentials vs. saturated calomel electrode (SCE)). Free energy change (ΔG_et_) for an electron transfer reaction was calculated from Equation (1) [109], where E_ox_, E_red_, E* and C represent the oxidation potential of the electron donor, the reduction potential of the electron acceptor, the excited-state energy level (determined from fluorescence experiments) and the coulombic term for the initially formed ion pair, respectively. Here, C is neglected as is usually the case for polar solvents.

ΔG_et_ = E_ox_ − E_red_ − E* + C (1)

### 2.6. UV-Visible Absorption, Steady-State Photolysis and Luminescence Experiments

Acquisition conditions have been detailed elsewhere [13,92,100].

### 2.7. Computational Procedure

Computational conditions have been detailed elsewhere [13,92,100,110,111]. Simulated absorption spectra of copper complexes were obtained by time-dependent density functional theory at the MPW1PW91/6-31G* level of theory on the relaxed geometries calculated at the UB3LYP/6-31G* level of theory.

### 2.8. Photocomposite Access Using a Near-UV Conveyor

Photocomposite materials were obtained using a Dymax-UV conveyor at 395 nm. Firstly, photosensitive resins were deposited on the glass fibers (reinforcement), then, this mixture was cured using an LED conveyor @395 nm (I = 4 W·cm^−2^). Distance between the belt and the LED was fixed to 15 mm, and the belt speed was fixed at 2 m/min.

### 2.9. Direct Laser Write (DLW) Experiment

The photosensitive formulation was deposited on a glass slide and 3D patterns were obtained under air using a computer-controlled diode laser at 405 nm (spot size = 50 μm). Analysis of the 3D patterns was carried out using a numerical optical microscope (DSX-HRSU from OLYMPUS Corporation, Rungis, France) [112].

## 3. Results

Light-absorption properties, initiation ability and applications (photocomposite synthesis and direct laser write) of the investigated copper complexes will be studied in this section.

### 3.1. Synthetic Routes to Copper Complexes Cu1 and Cu2

Copper complexes bearing a pyridine-based chelate ligand showed excellent photochemical properties for photocatalysis processes, such as a high-oxidation potential in the excited state [96,97,98,99], a long-excited-state lifetime, high-emission quantum yields and high-absorption properties in the UV-visible region. In this work, two new copper complexes have been developed, allowing, by the convenient choice of the ligands, a shift in the absorption properties in the visible range, while maintaining high efficiency.

To allow such a shift of the absorption properties towards the visible, two bipyridine ligands were synthesized starting from a chalcone. Structures of these chalcones and, therefore, of the corresponding ligands have been selected in order to induce a significant shift in the absorption spectrum of the copper complexes towards the visible range.

**Scheme 1:**
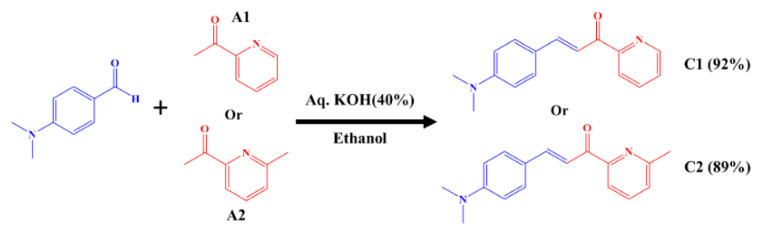
Synthesis and chemical structures of 2-carbonylpyridine-based chalcones.

For the synthesis of the two chalcones, a Claisen-Schmidt condensation reaction under basic conditions between 2-acetylpyridine A1 or 2-acetyl-6-methylpyridine A2 and aldehyde A3 was carried out (See Scheme 1) [113,114,115,116,117,118,119,120,121,122,123,124,125].

These two chalcones engage in a cyclization reaction with β-aminocrotonitrile, allowing the ligands to be formed. This reaction and its corresponding mechanism were described in the literature in 1992 by Masaki Matsui [105,106].

**Scheme 2:**
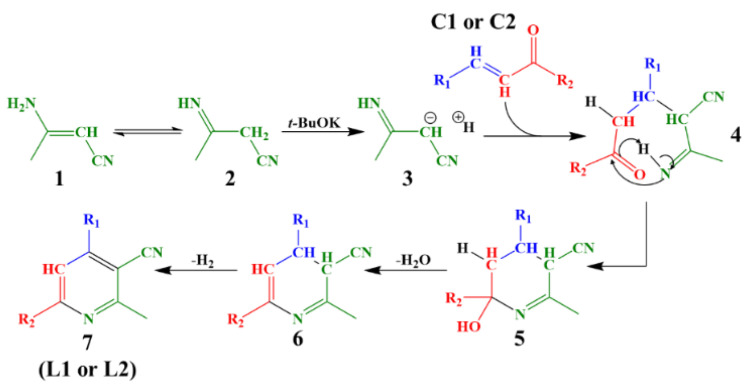
Probable mechanism described by Masaki Matsui.

As shown in Scheme 2, the mechanism proposed by Masaki Matsui involves the formation of two (L1 or L2) ligands. β-Aminocrotononitrile can exist as amino (1) and imino (2) isomers in solution. A Michael addition of thae carbanion of imino isomer 3 to C1 or C2 can give intermediate 4, which, after an intramolecular cyclization and dehydration reaction, can provide intermediate 6. At room temperature, dehydrogenation of 6 can give 7.

Starting from the conditions described by Masaki Matsui, the ligands were indeed obtained. The synthesis conditions were then optimized by using reduced quantities of solvent, base and β-aminocrotonitrile, and with a simplified reaction treatment due to the precipitation of the ligand at the end of the reaction, while maintaining a good yield. The optimized synthesis of the ligands is detailed in the synthetic procedure detailed above.

Heteroleptic copper complexes bearing a pyridine-based chelate ligand and a diphosphine ligand, such as 4,5-*bis*(diphenylphosphino)-9,9-dimethylxanthene (or Xantphos) and *bis*[2-(diphenylphosphino)phenyl]ether (or DPEphos), have previously been reported in the literature. In this work, this second ligand was replaced by a bisisonitrile ligand, namely *bis*(2-isocyanophenyl)phenylphosphonate. Indeed, as previously mentioned in a study reported by Matthias Knorn [107], copper complexes bearing this ligand showed a higher photocatalytic activity than their counterpart comprising a bisphosphine ligand. The lower activity of copper complexes comprising bisphosphine ligands can be assigned to the tendency of heteroleptic complexes to form an equilibrium with their homoleptic forms in solution [108], especially for heteroleptic copper complexes combining biphosphine and phenanthroline ligands. In contrast, in the case of the bisisonitrile ligand, studies have revealed the low ability of heteroleptic complexes to undergo ligand exchanges. The ligand was synthesized following the procedure described in the literature. Using these two ligands, the pyridine ligands and the bisisonitrile ligand in a one-step complexation reaction, the two desired copper complexes were obtained.

### 3.2. UV-Visible Absorption Spectra of Cu1 and Cu2

**Figure 3:**
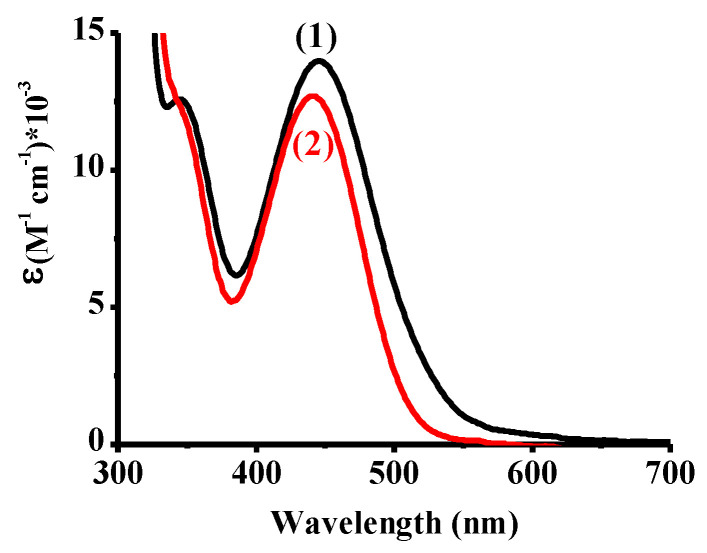
UV-visible absorption spectra of (**1**) Cu1 and (**2**) Cu2 in DCM.

**Figure 4:**
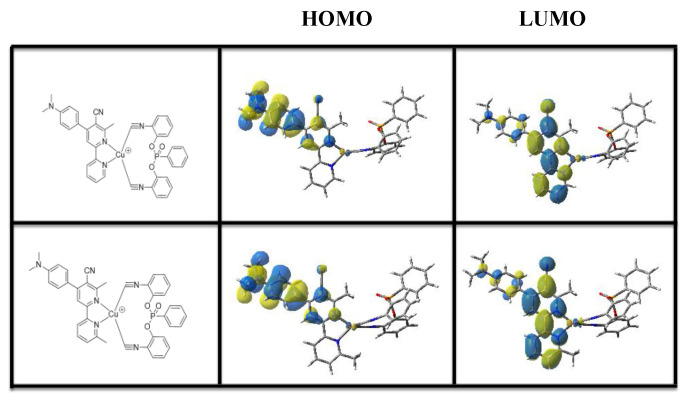
HOMO and LUMO for Cu1 and Cu2 at the UB3LYP/6–31G* level.

Ground-state absorption spectra of the new studied copper derivatives were determined in DCM and the results are presented in Figure 3. Extinction coefficients at different emission wavelengths used in photopolymerization experiments are reported in Table 1. New complexes are characterized by a broad absorption band, which extends between 380 nm and 650 nm, and high-extinction coefficients in the blue region, e.g., ε = 7570 M^−1^·cm^−1^, 7040 M^−1^·cm^−1^ @400 nm for Cu1 and Cu2, respectively. These compounds also have high-extinction coefficients at the emission wavelengths of LEDs (at 405 nm and 455 nm) used in different experiments achieved in this work, for example, ε_@405nm_ = 8460 and 7950 M^−1^·cm^−1^ for Cu1 and Cu2, respectively. Remarkably, a bathochromic shift in the absorption spectra of Cu2 was observed compared to that of Cu1. This effect could be related to the presence of a methyl group, which is considered as an electron-donating group (inductive effect), on the pyridine ligands of Cu2 (λ_max_ = 445 nm for Cu1 and 441 nm for Cu2). This difference could also be explained by the optimized geometries, as well as the frontier orbitals (highest occupied molecular orbital—HOMO—and lowest unoccupied molecular orbital—LUMO), which are shown in Figure 4.

### 3.3. Photopolymerization Experiments

#### 3.3.1. Free Radical Photopolymerization Using TA as a Benchmark Monomer

Due to their good light-absorption properties in the visible range, copper complexes were tested as photoinitiators for the FRP of acrylate-based monomers upon exposure to LEDs at 405 nm (I = 110 mW∙cm^−2^) and 455 nm (I = 75 mW∙cm^−2^).

**Figure 5:**
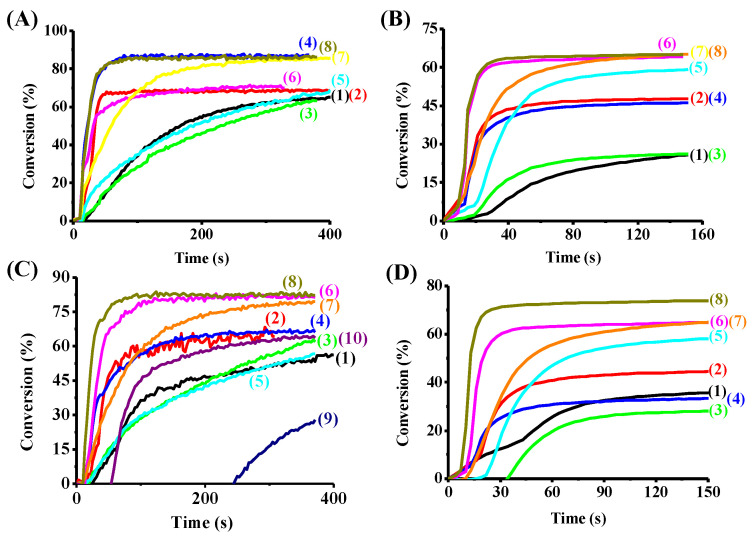
Free radical photopolymerization profiles of acrylate functions vs. irradiation time in: (**A**) thick samples @455 nm, (**B**) thin sample @455 nm, (**C**) thick sample @405 nm and (**D**) thin sample @405 nm: (1) Cu1/Iod (0.1%/1% *w*/*w*), (2) Cu2/Iod (0.1%/1% *w*/*w*), (3) Cu1/Iod (0.2%/1% *w*/*w*), (4) Cu2/Iod (0.2%/1% *w*/*w*), (5) Cu1/Iod/EDB (0.1%/1%/1% *w*/*w*/*w*), (6) Cu2/Iod/EDB (0.1%/1%/1% *w*/*w*/*w*), (7) Cu1/Iod/NPG (0.1%/1%/1% *w*/*w*/*w*), (8) Cu2/Iod/NPG (0.1%/1%/1% *w*/*w*/*w*), (9) Iod/EDB (1%/1% *w*/*w*) and (10) Iod/NPG (1%/1% *w*/*w*). Irradiation starts at *t* = 10 s.

In fact, photoinitiators (0.1% or 0.2%) were dissolved and mixed into the TA acrylate monomer in combination with Iod salt (1%) in order to make two-component photoinitiating systems, on the one hand, and in combination with Iod/amine (1%/1% *w*/*w*) to form three-component photoinitiating systems, on the other hand. Interestingly, these dyes exhibit excellent free radical photopolymerization conversions in thick and thin samples. The related results are gathered in Figure 5 and the data are summarized in Table 2. Remarkably, copper complexes alone, Iod and amine alone cannot polymerize the sample. Iod salt and amine are used as co-initiators in this work because they do not absorb visible light. It is important to introduce the dyes (i.e., the copper complexes) into the photosensitive formulations in order to obtain a good light absorption at 405 nm and 455 nm. The obtained results using copper derivatives in two-component PISs showed that Cu2 was more efficient than Cu1 for the FRP of TA using different PI percentages, e.g., FC ~ 64% for Cu1/Iod (0.1%/1% *w*/*w*) vs. 70% for Cu2/Iod (0.1%/1% *w*/*w*) (Figure 5A curve 1 vs. 2), and FC ~ 62% for Cu1/Iod (0.2%/1% *w*/*w*) vs. 85% for Cu2/Iod (0.2%/1% *w*/*w*) (Figure 5A curve 3 vs. 4).

Furthermore, Iod/NPG couple showed a weak polymerization initiation ability upon exposure to LEDs at 405 nm and 455 nm after 60 s (e.g., FC ~ 10% @405 nm). Interestingly, a greater efficiency was observed when NPG was incorporated into the formulation. Compared to their two-component system analogues, the different three-component PISs showed a better final conversion of reactive functions and a higher polymerization rate upon irradiation with LEDs at 405 nm or 455 nm (for example, an FC up to 86% is obtained with Cu1/Iod/NPG (0.1%/1%/1% *w*/*w*/*w*), and 88% using Cu2/Iod/NPG (0.1%/1%/1% *w*/*w*/*w*) with a LED @455 nm).

#### 3.3.2. Cationic Polymerization and IPN Synthesis

**Figure 6:**
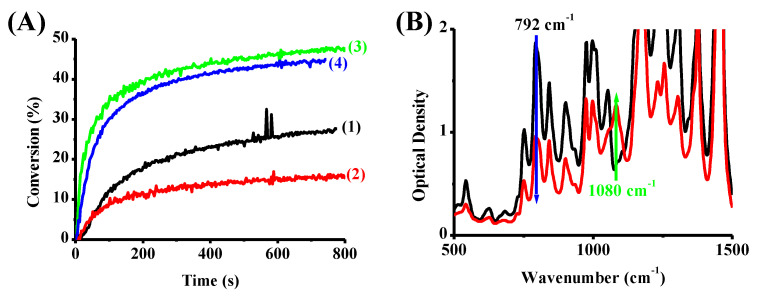
(**A**) Cationic polymerization (CP) profiles of epoxy functions (thin sample) vs. irradiation time upon exposure to LED at 405 nm: (1) Cu1/Iod (0.1%/1% *w*/*w*), (2) Cu2/Iod (0.1%/1% *w*/*w*), (3) Cu1/Iod/NVK (0.1%/2%/3% *w*/*w*/*w*) and (4) Cu2/Iod/NVK (0.1%/2%/3% *w*/*w*/*w*). (**B**) IR spectra recorded before and after polymerization. Irradiation starts at *t* = 10 s.

Typical epoxide function conversion-time profiles for Cu1 and Cu2-based photoinitiating systems are given in Figure 6 and the data are gathered in Table 3. In fact, the cationic polymerization of the epoxide functions was carried out under air and upon irradiation at 405 nm. Indeed, the cationic polymerization is insensitive to oxygen. As expected, copper complexes alone and the additives alone were not able to initiate the CP in these irradiation conditions. The addition of Iod salt or Iod/NVK into the formulation containing the PI induced good photopolymerization profiles, i.e., the combination Cu/Iod/NVK (0.1%/2%/3% *w*/*w*/*w*) is very efficient to produce polymer materials in terms of Rp and final epoxy function conversion compared to Cu/Iod (0.1%/1% *w*/*w*), e.g., (FC ~ 50% for Cu1/Iod/NVK (0.1%/2%/3% *w*/*w*/*w*) vs. 27% for Cu1/Iod (0.1%/1% *w*/*w*)). The consumption of epoxide functions was accompanied by the formation of a polyether network (appearance of peak at ~1080 cm^−1^), characterizing the obtained polymer.

**Figure 7:**
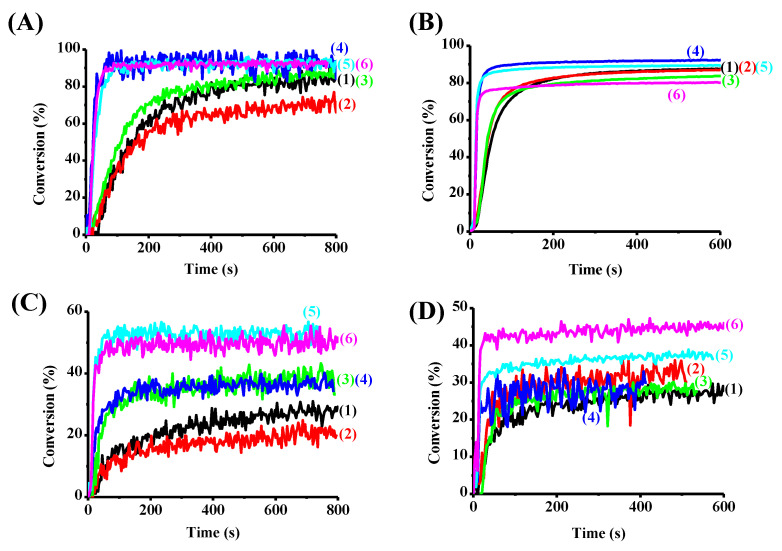
Photopolymerization profiles of acrylate functions (**A**) (thick sample), (**B**) (thin sample)) upon irradiation at 455 nm, and epoxide function ((**C**) (thick sample), (**D**) (thin sample)) upon exposure to LED at 405 nm of TA/EPOX using Cu1/Iod/NPG (0.1%/1%/1% *w*/*w*/*w*): (1) 30%/70%, (2) 50%/50%, (3) 70%/30%, and Cu2/Iod/NPG (0.1%/1%/1% *w*/*w*/*w*): (4) 30%/70%, (5) 50%/50%, (6) 70%/30%. The irradiation starts at *t* = 10 s.

On the other hand, IPNs syntheses were also carried out in this work and polymerization tests were performed in thick and thin samples using LEDs at 405 nm and 455 nm (See Table 4 and Table 5). Photopolymerization profiles for the IPN formation are presented in Figure 7. For example, the acrylic network formation was very fast with a high final conversion (98%) for Cu2/Iod/NPG (0.1%/1%/1% *w*/*w*/*w*) in TA/EPOX (50%/50%) upon irradiation at 455 nm, and the formation of the epoxy network was also efficient (high final conversion and Rp) using this system (FC ~ 55%).

### 3.4. Photocomposites Synthesis

Nowadays, many of our modern technologies require materials with enhanced properties. This is particularly true for materials used in aerospace, underwater and transportation applications. For example, for aeronautical applications, engineers research materials with properties of low density, rigid, solid, impact resistance, temperature and pressure resistance and obviously materials that do not easily corrode. For this purpose, composite materials have been used for different applications. By definition, a composite material is composed of a least two components that results in better properties than those of the individual components used alone: matrix (monomer blends) and reinforcement. The main advantages of composite materials are their high stiffness, strength and low density. The introduction of light for the synthesis of photocomposites will make the manufacture of these materials more ecological.

**Figure 8:**
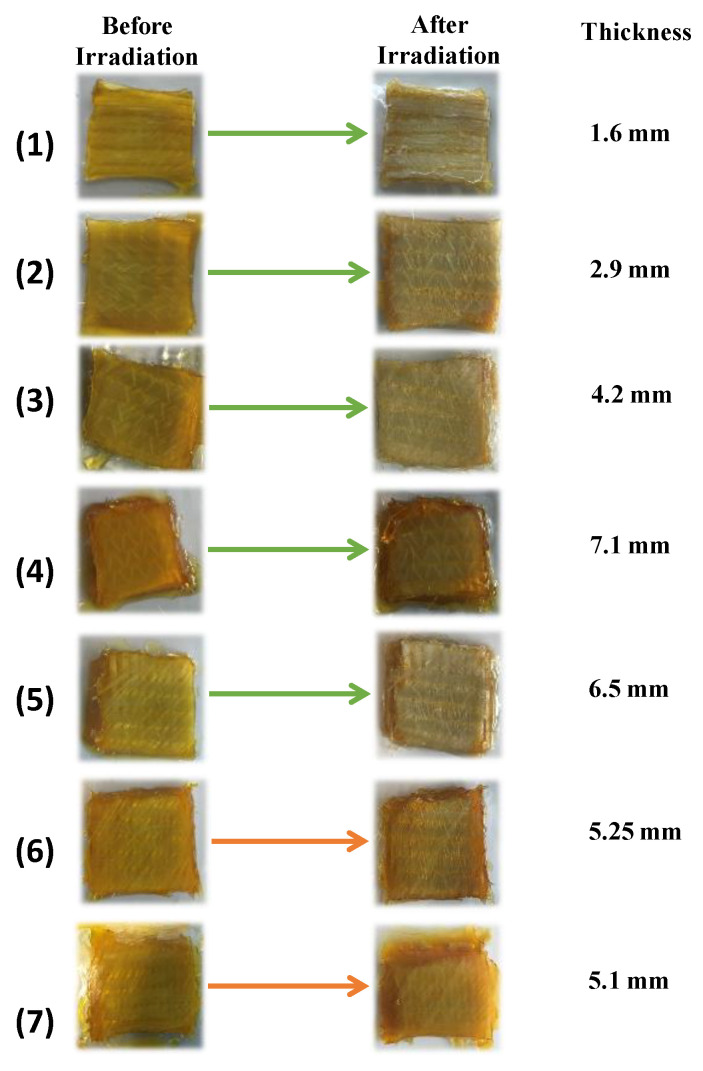
Free radical photopolymerization for the photocomposite synthesis upon near-UV irradiation at 395 nm (4 W·cm^−2^) using Cu2/Iod/NPG (0.1%/1%/1% *w*/*w*/*w*) in TA [(1)–(4)], (5)) Cu2/Iod/NPG (0.1%/1%/1% *w*/*w*/*w*) in TMPTA, (6) Cu1/Iod/NPG (0.1%/1%/1% *w*/*w*/*w*) in TMPTA and (7) Cu1/Iod/NPG (0.1%/1%/1% *w*/*w*/*w*) in TA.

In this study, the matrix is based on acrylic monomers, such as TMPTA or TA, and the second component (reinforcement) is based on glass fibers. Firstly, the acrylic resins were deposed on the reinforcement (50%/50% *w*/*w*) and the mixtures were irradiated using an LED conveyor at 395 nm. Interestingly, a very fast polymerization on the surface and the bottom was observed with tack-free surfaces, after one pass only using one layer of glass fibers (1 mm). Increasing the reinforcement thickness by adding several layers, the polymerization on the surface is always fast and takes place after one pass, but the curing on the bottom is more complicated and will be done after several passes using Cu1 or Cu2/Iod/NPG (0.1%/1%/1% *w*/*w*/*w*) as PISs. The curing photocomposite results are depicted in Figure 8 and Table 6.

### 3.5. Direct Laser Write (DLW)

**Figure 9:**
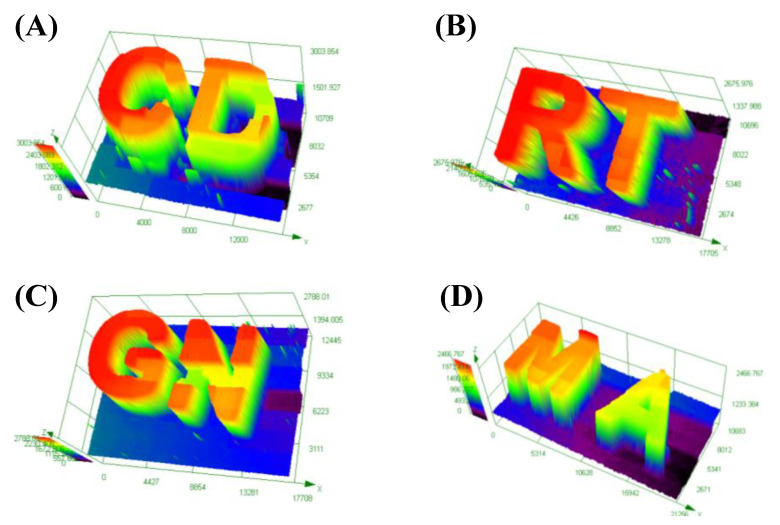
3D patterns produced by free radical photopolymerization of acrylate functions (TA or TMPTA) using a laser diode at 405 nm and their characterization by numerical optical microscopy: (**A**) Cu2/Iod/TMA (0.057%/1%/0.46% *w*/*w*/*w*) in TA, (**B**) Cu1/Iod/TMA (0.058%/0.506%/0.44% *w*/*w*/*w*) in TMPTA; (**C**) Cu2/Iod/TMA (0.048%/0.65%/0.259% *w*/*w*/*w*) in TA and (**D**) Cu1/Iod/TMA (0.05%/0.5%/0.305% *w*/*w*/*w*).

The new copper complexes were tested in some direct laser write experiments for the FRP of TMPTA or TA using a laser diode at 405 nm (spot size: 50 μm). The obtained 3D patterns were carried out under air and using different PISs based on Cu1/Iod/TMA, Cu2/Iod/TMA in TA or TMPTA (Figure 9). Due to their high ability to initiate the FRP of acrylates, these systems were able to generate high-spatial-resolution 3D patterns with a great thickness of curing (~2500 μm) in the irradiated area. As such, 3D patterns were generated with very short irradiation times (2–3 min) and they were characterized by numerical microscopy.

### 3.6. Mechanical Properties: Tensile Test Measurements

The tensile strength of IPNs synthesized using different compositions of the TA/EPOX mixture are presented in Table 7. The results show that with the increase in the percentage of acrylic monomer, the tensile strength increases, which may be due to the rigid character of the acrylates (e.g., 7.2 MPa for Cu2/Iod/NPG in TA/EPOX (30%/70%) vs. 37.2 MPa for the same system in TA/EPOX (70%/30%)).

## 4. Discussion

In order to explain the initiating ability of the organometallic complexes, their photochemical and photophysical properties were studied using different characterization techniques, allowing for the characterization of the associated chemical mechanisms.

### 4.1. Steady-State Photolysis of the Investigated Compounds

**Figure 10:**
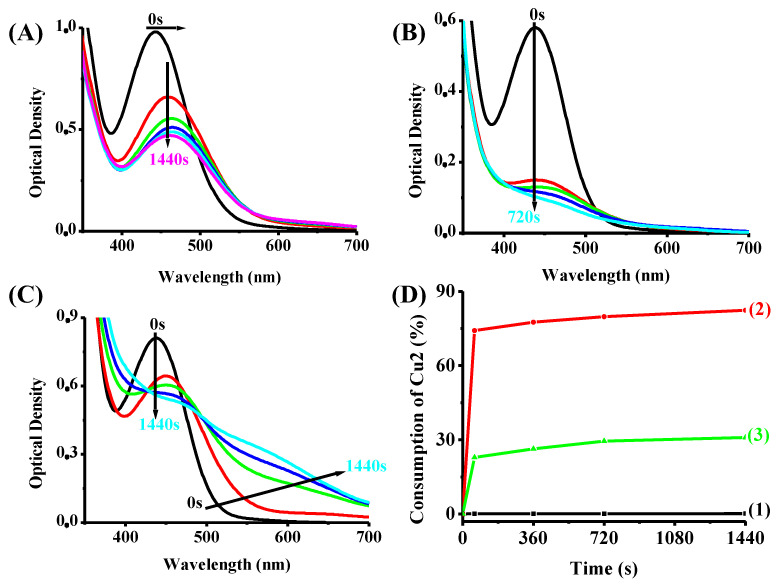
Photolysis experiments of (**A**) Cu1 with Iod salt (10^−2^ M), (**B**) Cu2 with Iod salt (10^−2^ M) and (**C**) Cu2/Iod/NPG (10^−2^ M) upon exposure to LED at 405 nm. (**D**) Consumption percentage of Cu2: (1) alone, (2) with Iod, (3) with Iod/NPG.

**Figure 11:**
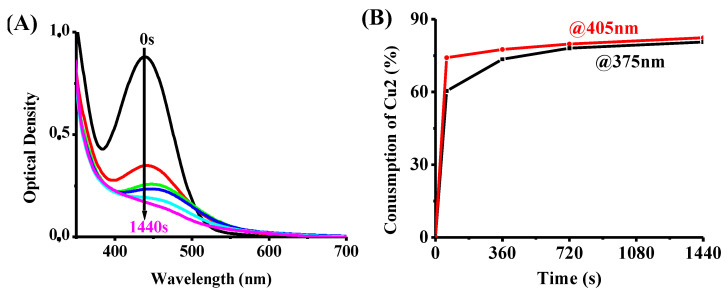
(**A**) Photolysis of Cu2 with Iod (10^−2^ M) upon irradiation @375 nm. (**B**) Consumption of Cu2 with irradiation @375 nm and 405 nm.

Photolyses of Cu1 and Cu2 dyes in DCM were investigated upon irradiation at 375 nm and 405 nm, and the related results are shown in Figure 10. First of all, no photolysis occurred for Cu1 and Cu2 alone (0% consumption) upon irradiation at 375 nm and 405 nm, but the incorporation of the iodonium salt into the photosensitive solution could promote the degradation of the dyes, so that a strong decrease in the absorbance band intensity was observed by increasing the irradiation time, e.g., consumption ~ 80% @375 nm and 82% for Cu2/Iod at 375 nm and 405 nm, respectively (Figure 11B). It is important to note that the photolysis of Cu1 in the presence of Iod salt involved the formation of a photoproduct after 60 s of irradiation in the solution, which had an absorption band more shifted in the visible (bathochromic effect) spectrum, then this photoproduct degrades under the effect of the irradiation (Figure 10A).

This difference between these two consumption percentages may be due to the high light-absorption ability of Cu2 at 405 nm, as well as the highest intensity of the LED at 405 nm (110 mW·cm^−2^), compared to that at 375 nm (75 mW·cm^−2^). Furthermore, in the case of three-component PISs, the consumption of Cu2 was lower compared to that of the two-component Cu2/Iod system. It can be confidently assigned to the regeneration of Cu2 in the three-component system due to the presence of the sacrificial amine or the formation of new photoproducts (%consumption ~ 31%).

### 4.2. Photoluminescence and Electrochemical Properties

**Figure 12:**
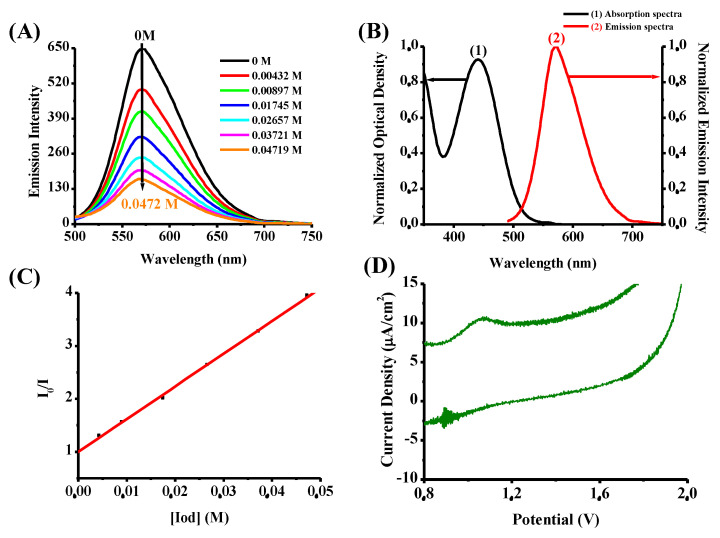
(**A**) Fluorescence quenching of Cu2 by Iod salt, (**B**) E_S1_ determination for Cu2, (**C**) Stern–Volmer coefficient determination of Cu2 quenched by Iod and (**D**) oxidation potential determination of Cu2.

Fluorescence emission spectra, fluorescence quenching (measured using a JASCO FP-6200 spectrofluorimeter, Lisses, France) and the oxidation potential (measured in DCM by cyclic voltammetry, OrigaLys, Rillieux-la-Pape, France) results of the different Cu derivatives are gathered in Figure 12 and Table 8. The excited-state energy was calculated from the crossing point of the emission and absorption spectra. Using these different values, the free-energy change (ΔG) could be calculated; this parameter reflects the reactivity between Cu and Iod. In fact, a slight decrease in the fluorescence intensity was observed for Cu1/Iod, but this emission spectra showed a strong decrease for Cu2 upon addition of Iod. These behaviors explain the high reactivity of Cu2/Iod compared to Cu1/Iod e.g., ϕ = 0.55 for Cu1/Iod vs. 0.74 for Cu2/Iod. In addition, the ΔG value is negative for both complexes, so that the photo-oxidation interaction Cu1 (or Cu2)/Iod is favorable in both cases, with a superiority observed for Cu2 (ΔG = −0.74 and 0.62 eV for Cu2 and Cu1, respectively). It, therefore, explains the high-photoinitiation ability of Cu2 compared to Cu1.

**Scheme 3:**
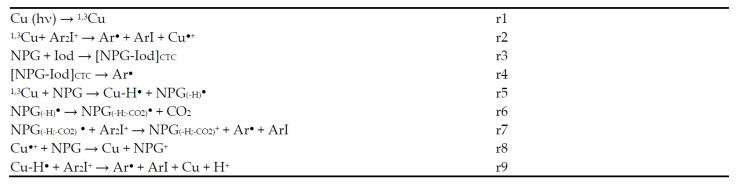
Proposed chemical mechanisms.

Finally, the initiation ability of the new copper complexes could be explained by different characterization techniques, which allowed us to propose a chemical photoinitiation mechanism. Firstly, Cu is excited upon irradiation at 405 or 455 nm and interacts with Iod to generate aryl radical (Ar^●^) and radical Cu^●+^ [r1–r2]. A charge transfer complex CTC can be formed after adding NPG into the photosensitive formulation. This complex is able to produce aryl radicals as active species for the radical photopolymerization [r3–r4]. Then, ^1,3^Cu could react with NPG and generate two radicals (NPG_-H_^●^, Cu-H^●^) [r5], and the first radical can undergo a decarboxylation and produce active radicals (NPG_(-H,-CO2)_^●^) [r6]. This radical can also lead to the formation of two active species after interaction with Iod salt (NPG_(-H,-CO2)_^+^, Ar^●^) [r7]. Lastly, copper complex derivatives are regenerated [r8–r9] (See Scheme 3).

## 5. Conclusions

In the present paper, new copper complexes were synthesized and tested as photoinitiators. These compounds have strong visible-light absorption and are able to initiate both the free radical photopolymerization and cationic polymerization. IPN synthesis through the simultaneous polymerization of acrylate/epoxy monomer blends was performed under air upon irradiation at 405 nm and 455 nm, using a very low quantity of copper complex, in two or three-component PIS. Cu2 showed a very interesting photoinitiation capacity compared to Cu1 in terms of final conversions of reactive functions and polymerization rates. The high reactivity of these compounds was demonstrated through some direct laser write experiments, where high-spatial-resolution 3D patterns were obtained. In addition, the synthesis of thick glass fiber photocomposites was possible. This work paves the way for the development of new organometallic photoinitiators.

## Figures and Tables

**Table 1:** Maximum absorption wavelengths (λ_max_), extinction coefficients at λ_max_, and molar extinction coefficients for the investigated copper complexes at different emission wavelengths for different LEDs used.

	λ_max_ (nm)	ε_max_ (M^−1^∙cm^−1^)	ε_375nm_ (M^−1^∙cm^−1^)	ε_405nm_ (M^−1^∙cm^−1^)	ε_455nm_ (M^−1^∙cm^−1^)
Cu1	445	14,000	7150	8460	13,600
Cu2	441	12,700	5760	7950	11,740

**Table 2:** Final reactive functions conversion (FC) for TA monomer using two or three-component PISs upon exposure at different wavelengths (LED at 405 and 455 nm).

	At 405 nm				At 455 nm			
	Thick Sample		Thin Sample		Thick Sample		Thin Sample	
Cu1/Iod	56% ^a^	62% ^b^	36% ^a^	28% ^b^	64% ^a^	64% ^b^	26% ^a^	26% ^b^
Cu2/Iod	64% ^a^	67% ^b^	45% ^a^	33% ^b^	70% ^a^	87% ^b^	48% ^a^	46% ^b^
Cu1/Iod/amine	57% ^c^	80% ^d^	58% ^c^	65% ^d^	69% ^c^	85% ^d^	59% ^c^	65% ^d^
Cu2/Iod/amine	82% ^c^	83% ^d^	65% ^c^	74% ^d^	71% ^c^	87% ^d^	64% ^c^	65% ^d^

**^a^**: Cu/Iod (0.1%/1% *w*/*w*), **^b^**: Cu/Iod (0.2%/1% *w*/*w*), **^c^**: Cu/Iod/EDB (0.1%/1%/1% *w*/*w*/*w*), **^d^**: Cu/Iod/NPG (0.1%/1%/1% *w*/*w*/*w*).

**Table 3:** Final reactive function conversions for EPOX monomer using two and three-component PISs upon irradiation at 405 nm during the polymerization of thin samples.

	Cu/Iod (0.1%/1% *w*/*w*)	Cu/Iod/NVK (0.1%/2%/3% *w*/*w*/*w*)
Cu1	27%	49%
Cu2	16%	45%

**Table 4:** Final conversions of acrylate and epoxides functions for the IPN synthesis of TA/EPOX blend using Cu1 (or Cu2)/Iod/NPG (0.1%/1%/1%/ *w*/*w*/*w*) as PIS upon exposure to visible light at 405 nm.

	IPN Synthesis of TA/EPOX Blend Performed in Thick Sample at 405 nm			IPN Synthesis of TA/EPOX Blend Performed in Thin Sample at 405 nm		
	30%/70%	50%/50%	30%/70%	30%/70%	50%/50%	70%/30%
Cu1	90%/25%	90%/15%	93%/27%	88%/25%	87%/31%	84%/47%
Cu2	99%/30%	98%/20%	96%/38%	92%/22%	90%/35%	80%/32%

**Table 5:** Final conversions of acrylate and epoxides functions for the IPN synthesis of TA/EPOX blend using Cu1 (or Cu2)/Iod/NPG (0.1%/1%/1%/ *w*/*w*/*w*) as PIS upon exposure to visible light at 455 nm.

	IPN Synthesis of TA/EPOX Blend Performed in Thick Sample @455 nm			IPN Synthesis of TA/EPOX Blend Performed in Thin Sample @455 nm		
	30%/70%	50%/50%	30%/70%	30%/70%	50%/50%	70%/30%
Cu1	90%/30%	90%/23%	90%/41%	90%/15%	90%/25%	90%/15%
Cu2	100%/38%	98%/55%	99%/51%	98%/22%	99%/30%	98%/22%

**Table 6:** Photocomposite synthesis results using TA as acrylic monomer (or TMPTA) and number of passes to reach the tack-free character on the surfaces.

	Thickness	Number of Passes to Reach Tack-Free Character on the Surface	Number of Passes to Reach Tack-Free Character on the Bottom
Cu2/Iod/NPG	1.6 mm	1	1
Cu2/Iod/NPG	2.9 mm	1	2
Cu2/Iod/NPG	4.2 mm	1	6
Cu2/Iod/NPG (TMPTA)	7.1 mm	1	25
Cu2/Iod/NPG	6.5 mm	1	30
Cu1/Iod/NPG	5.25 mm	1	40
Cu1/Iod/NPG (TMPTA)	5.1 mm	1	45

**Table 7:** The tensile strength of IPN materials as a result of hybrid polymerization of the TA/EPOX mixture using Cu/Iod/NPG (0.1%/1%/1% *w*/*w*/*w*) as a photoinitiating system.

Tensile Strength [MPa]	0.1%PA/Iod/NPG TA/EPOX (30% 70%) @ 395 nm	0.1% PA/Iod/NPG TA/EPOX (50% 50%) @ 395 nm	0.1% PA/Iod/NPG TA/EPOX (70% 30%) @ 395 nm
Cu1	6.5	7.3	34.3
Cu 2	7.2	26.3	37.2

**Table 8:** Parameters characterizing the chemical mechanisms between Cu1 (or Cu2) and Iod. For Iod, a reduction potential of −0.7 eV was used for the ∆Get calculations.

	E_ox_ (V)	E_S1_ (eV)	ΔG_(Cu/Iod)_ (eV)	Κ_SV(Cu/Iod)_	Φ_(Cu/Iod)_	E_ox_ (V)
Cu1	1.07	2.34	−0.62	13.58	0.55	Cu1
Cu2	0.97	2.41	−0.74	61.83	0.74	Cu2

## Data Availability

Not applicable.
